# Hdac3, Setdb1, and Kap1 mark H3K9me3/H3K14ac bivalent regions in young and aged liver

**DOI:** 10.1111/acel.13092

**Published:** 2019-12-19

**Authors:** Andrew J. Price, Mohan C. Manjegowda, Jessica Kain, Swetha Anandh, Irina M. Bochkis

**Affiliations:** ^1^ Department of Pharmacology University of Virginia Charlottesville VA USA

**Keywords:** bivalent domain, chromatin remodeling, Hdac3, Kap1, lipid metabolism, liver, Setdb1

## Abstract

Post‐translational modifications of histone tails play a crucial role in gene regulation. Here, we performed chromatin profiling by quantitative targeted mass spectrometry to assess all possible modifications of the core histones. We identified a bivalent combination, a dually marked H3K9me3/H3K14ac modification in the liver, that is significantly decreased in old hepatocytes. Subsequent sequential ChIP‐Seq identified dually marked single nucleosome regions, with reduced number of sites and decreased signal in old livers, confirming mass spectrometry results. We detected H3K9me3 and H3K14ac bulk ChIP‐Seq signal in reChIP nucleosome regions, suggesting a correlation between H3K9me3/H3K14ac bulk bivalent genomic regions and dually marked single nucleosomes. Histone H3K9 deacetylase Hdac3, as well as H3K9 methyltransferase Setdb1, found in complex Kap1, occupied both bulk and single nucleosome bivalent regions in both young and old livers, correlating to presence of H3K9me3. Expression of genes associated with bivalent regions in young liver, including those regulating cholesterol secretion and triglyceride synthesis, is upregulated in old liver once the bivalency is lost. Hence, H3K9me3/H3K14ac dually marked regions define a poised inactive state that is resolved with loss of one or both of the chromatin marks, which subsequently leads to change in gene expression.

## Graphical abstract

Model describing establishment of H3K9me3/H3K14ac bivalent mark. In the first step, close residues H3K9 and H3K14 are acetylated by the same histone acetyltransferase (HAT). Then, H3K9 is deacetylated by Hdac3, allowing for subsequent triple methylation of H3K9 by Setdb1, which is found in complex with Kap1, and establishment of the bivalent region.

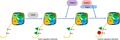

## 1 INTRODUCTION

Post‐translational modifications of histone tails play a crucial role in gene regulation (Jenuwein & Allis, 2001). In particular, tri‐methylation of histone H3 lysine 9 (H3K9) is associated with heterochromatin formation (Saksouk, Simboeck, & Dejardin, 2015) while acetylation of histone H3 lysine 14 (H3K14) is critical for DNA damage and checkpoint activation and circadian regulation (Wang et al., 2012) (Tasselli & Chua, 2015). Typically, acetylation of H3K9 and H3K14 co‐occur together, leading to gene activation (Karmodiya, Krebs, Oulad‐Abdelghani, Kimura, & Tora, 2012). However, levels H3K9me3 and H3K14ac increased together in livers of offspring of mothers fed high‐fat diet (Suter et al., 2014), leading to metabolic changes.

A specific histone modification pattern of two co‐occurring marks, one activating and the other repressing gene expression, named “bivalent domain” was first described in mESCs (Bernstein et al., 2006). These dually marked H3K4me3/H3K27me3 regions were in a poised inactive state, with one mark transmitted to one cell type, leading to gene activation, and the other to another cell type, leading to gene repression, upon differentiation. The role of bivalent domains in differentiated tissues is less clear. Presence of another bivalent mark, consisting of H3K9me3 and H3K14ac modifications, has been recently reported in mESCs where the authors demonstrate that the Tudor domain of Setdb1 recognized H3K14ac, whereas the SET domain of Setdb1 methylated H3K9me3 (Jurkowska et al., 2017). This study linked Setdb1 binding at these bivalent domains to silencing of LINE elements.

We have recently connected epigenetic changes to metabolic alterations in aged liver, finding that both altered nucleosome occupancy and redistribution of lamina‐associated domains are correlated with development of hepatic steatosis (Bochkis, Przybylski, Chen, & Regev, 2014; Whitton et al., 2018).

Since histone modification patterns are altered in many organisms with aging (Sidler, Kovalchuk, & Kovalchuk, 2017), we set out to investigate which chromatin marks were altered in old hepatocytes. Chromatin profiling by quantitative target mass spectrometry identified a bivalent H3K9me3/H3K14ac mark in the liver, which is significantly decreased in old hepatocytes. Subsequent sequential ChIP‐Seq identified dually marked single nucleosome regions, with reduced number of sites and decreased signal in old livers, confirming mass spectrometry results. We detected H3K9me3 and H3K14ac bulk ChIP‐Seq signal in reChIP single nucleosome regions, suggesting a correlation between H3K9me3/H3K14ac bulk bivalent genomic regions and dually marked single nucleosomes. Histone H3K9 deacetylase Hdac3, as well as H3K9 methyltransferase Setdb1, found in complex Kap1, occupied both bulk and single nucleosome bivalent regions in both young and old livers, correlating to presence of H3K9me3. Expression of genes associated with bivalent regions in young liver, including those regulating cholesterol secretion and triglyceride synthesis, is upregulated in old liver once the bivalency is lost. Hence, H3K9me3/H3K14ac dually marked regions define a poised inactive state that is resolved with loss of one or both of the chromatin marks, which subsequently leads to change in gene expression.

## 2 RESULTS

**Figure 1:**
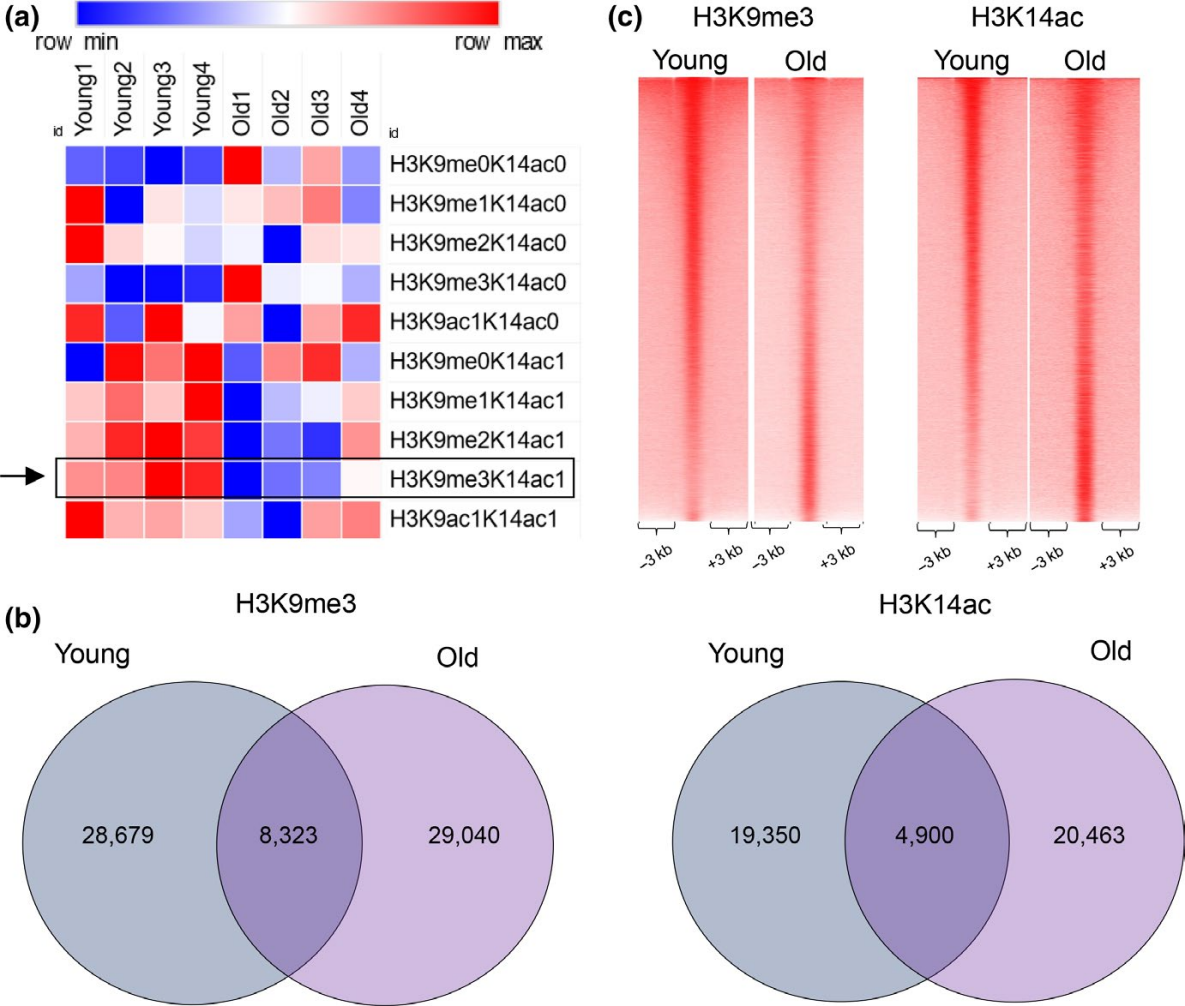
New bivalent chromatin state identified by proteomic analysis in mammalian liver. (a) Heatmap showing the relative intensities of histone marks determined by targeted mass spectrometry of histone tails in young and old livers (values are normalized to row's median). A bivalent H3K9me3/H3K14ac mark (marked with a rectangle and an arrow) was identified in both young and old livers and was quantitatively reduced in old livers. (b) Venn diagram showing the results of genome‐wide location analysis for H3K9me3 (left panel) and H3K14ac (right panel) marks in young and old liver. For H3K9me3, Peak‐Seq identified 28,679 sites in young liver and 29,030 in old liver, with 8,323 in the overlap. For H3K14ac, 19,350 peaks in young liver and 20,463 peaks in old liver were called, with 4,900 in the overlap. (c) Heatmaps comparing H3K9me3 (left panel) and H3K14ac (right panel) ChIP‐Seq coverage in young and old livers. Overall, there was more H3K9me3 but less H3K14ac signal in young livers as compared to the old. Reads were merged from two replicates for H3K9me3 and H3K14ac ChIP‐Seq data in both conditions. Data for H3K9me3 were accessed from our previous study (Whitton et al., 2018)

*H3K9me3/H3K14ac bivalent chromatin state identified by proteomic analysis in mammalian liver.* We have previously reported that changes in nucleosome occupancy are associated with metabolic dysfunction in aged livers (Bochkis et al., 2014). In addition, numerous modifications of histone tails have been altered with aging in many cell types (Sidler et al., 2017). Hence, we decided to investigate all post‐translational modifications on histone tails in an unbiased manner to determine which chromatin marks change in aged fatty liver (Table S1). Chromatin profiling by quantitative targeted mass spectrometry (Creech et al., 2015) targeted both individual and combinations of chromatin modifications that resided on the same histone tail, identifying co‐occurrence of methylated lysine 9 and acetylated lysine 14 (K9mex/K14ac, x: 1–3) on histone H3 tails from young (3 months) and old (21 months) mouse livers. Presence of these bivalent modifications quantitatively decreased in old livers (Figure 1a). Although the three combinations followed a similar trend, difference in abundance of H3K9me3/K14ac peptides in young and old livers is most significant (*p*‐values < .024, .007, .002 for H3K9me1, H3K9me2, and H3K9me3, respectively). We decided to investigate this dual mark for two reasons. First, changes in heterochromatin, marked by H3K9me3, were implicated to drive human aging (Zhang et al., 2015). And second, hepatic levels of both H3K9me3 and H3K14ac increased in offspring of mothers fed high‐fat diet (Suter et al., 2014), suggesting these modifications are correlated with metabolic changes.

**Figure 2:**
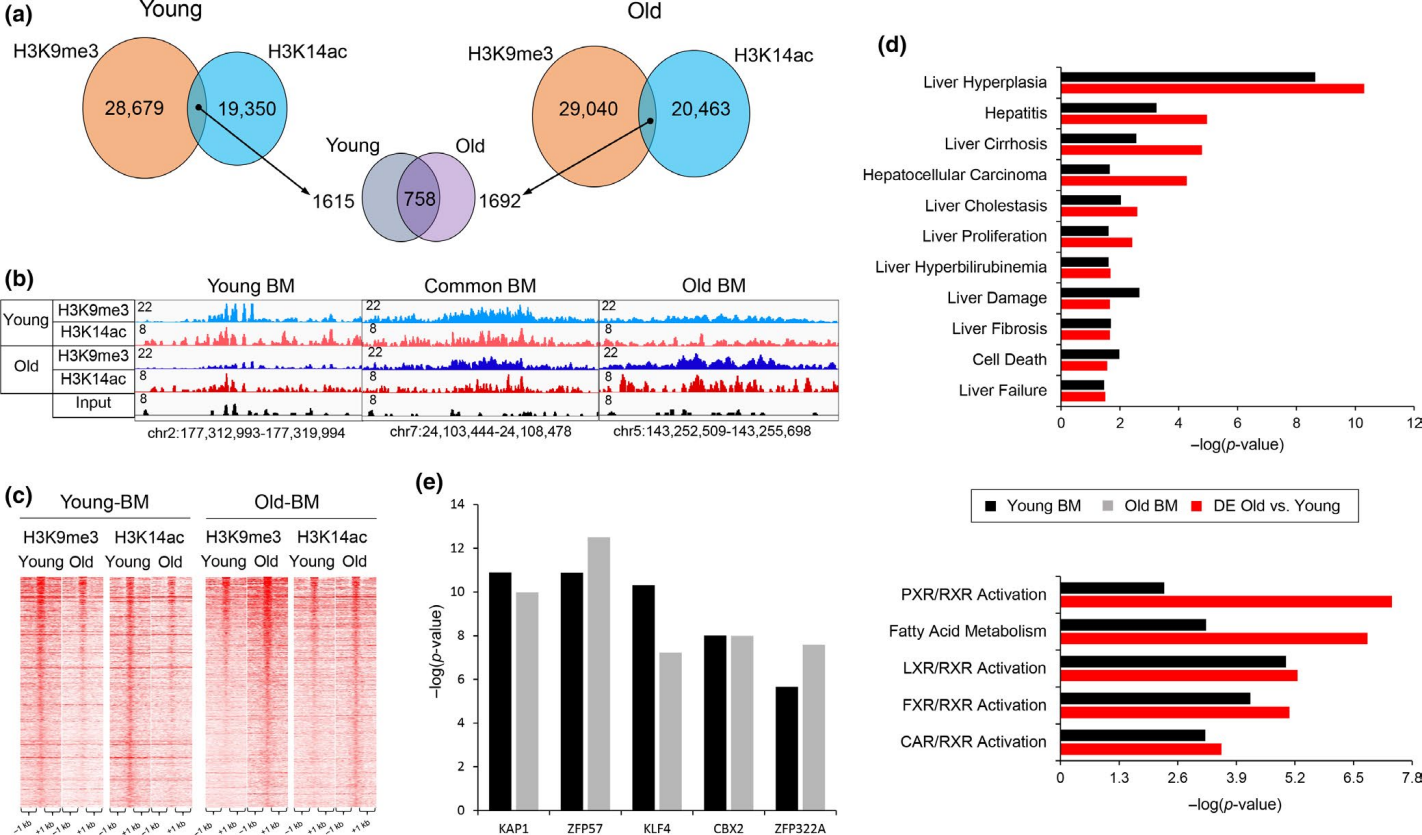
Bivalent regions in young are distinct from those of aged liver. (a) Venn diagrams showing the overlap of H3K9me3 and H3K14ac marks in young (left panel) and old (right panel) livers, identifying 1615 and 1692 bivalent regions (H3K9me3/H3K14ac) with 758 domains were present at both conditions (middle panel). (b) Examples of genomic regions with H3K9me3/H3K14ac bivalent mark specific to young liver (left panel: chr2:177,312,993–177,319,994, bivalent region in red rectangle), common to both (middle panel: chr7:24,103,444– 24,108,478) and old livers (right panel: chr5:143,252,509–143,255,698, bivalent region in red rectangle). Magnitude of the ChIP‐Seq signal is shown on y‐axis. (c) Heatmaps showing H3K9me3 and H3K14ac ChIP‐Seq signal at bivalent regions in young (left panel) and in old livers (right panel). (d) Comparison of overrepresented disease functions (top bar graph) and pathways (bottom bar graph) identified by Ingenuity Pathway Analysis of genes associated with bivalent mark in young livers (Young BM black bar) and differentially regulated genes in aged livers (DE Old vs. Young, red bar). Genes associated with bivalent regions are identified by Genomic Regions Enrichment of Annotations Tool (GREAT, see methods for details). (e) Chromatin‐x Enrichment Analysis (ChEA 2016, part of Enrichr gene set enrichment analysis (Kuleshov et al., 2016) of bivalent regions of young (Young BM, black bar) and old (Old BM, gray bar) livers by Enrichr shows Kap1 (*p*‐value young: 2.89 × 10^–11^, old: 7.55 × 10^–10^) binding sites are most significantly enriched at these bivalent regions. Reads are merged from two replicates in each condition. ChIP‐Seq data for H3K9me3 (Whitton et al., 2018) and RNA‐Seq data (Bochkis et al., 2014) for differentially regulated genes in aged liver are from our previous studies

Next, we performed genome‐wide location analysis (ChIP‐Seq) of H3K9me3 (previous study (Whitton et al., 2018)) and H3K14ac to determine the genomic regions marked by the dual modification. We identified 28,679 peaks in young and 29,040 peaks in the old livers marked by H3K9me3 (peak calls by PeakSeq, FDR < 5%, *q*‐value = 0.01), with an overlap of 8,323 sites called bound by PeakSeq at this threshold. Similar analysis established 19,350 peaks in young and 20,463 peaks in old livers marked by H3K14ac (FDR < 5%, *q*‐value = 0.02), with an overlap of 4,900 sites called bound by PeakSeq at this threshold (Figure 1b). Although the number of peaks called at the chosen threshold was similar, there was more H3K9me3 but less H3K14ac signal in young livers as compared to the old (Figure 1c). Next, comparing binding regions for both marks, we identified 1,615 bivalent genomic sites in young and 1,692 in old livers, with 758 in common (Figure 2a, examples in 2b). The number of comparable bivalent genomic regions observed in young and old livers is in contrast to mass spectrometry data showing a quantitative decrease in this bivalent modification on a single histone tail (Figure 1a).

Bivalent regions specific to young livers lose both marks in old hepatocytes. Similarly, bivalent regions specific to old livers are not marked by both modifications in young livers (Figure 2c). Next, we mapped regions with bivalent marks to closest genes using ChIP‐Seq GREAT (McLean et al., 2010) and compared them to differentially expressed transcripts in old livers (RNA‐Seq) (Bochkis et al., 2014) using Ingenuity Pathway Analysis (IPA). There are many similarities between genes residing in dually marked regions in young livers and genes whose expression changes in old livers, with nuclear receptor (LXR, FXR, CAR, and PXR) activation pathways comparably enriched (Figure 2d). This comparison suggests that genes in H3K9me3/H3K14ac bivalent regions are in poised inactive state in adult liver, similar to classical H3K4me3/H3K27me3 bivalent domains characterized in mESCs (Bernstein et al., 2006). Genes regulated by nuclear receptor gene pathways in bivalent regions in young livers get activated in old hepatocytes.

In contrast, only several pathways are similar between genes mapped from dually marked regions in old livers and differentially expressed genes (“Oxidative Stress,” *p*‐value <8.51E‐3, “Liver Proliferation,” *p*‐value < 8.51E‐3, “Ahr signaling,” *p*‐value < 9.77E‐3). Additional functional analysis using EnrichR (Kuleshov et al., 2016) identified Kap1 binding sites (ChIP‐Seq, http://www.ncbi.nlm.nih.gov/geo/query/acc.cgi?acc=GSE31183) as significantly overrepresented among both regions identified as dually marked in both young and old livers (*p*‐values < 7.55E‐10 and < 2.90E‐11, respectively, Figure 2e). Kap1 interacts with Setdb1 (Schultz, Ayyanathan, Negorev, Maul, & Rauscher, 2002), a chromatin regulator that is associated with H3K9me3/H3K14ac dually marked regions in mESCs (Jurkowska et al., 2017). Our analysis suggests that Setdb1 in complex with Kap1 is also associated with H3K9me3/H3K14ac bivalent regions in young and old livers.

**Figure 3:**
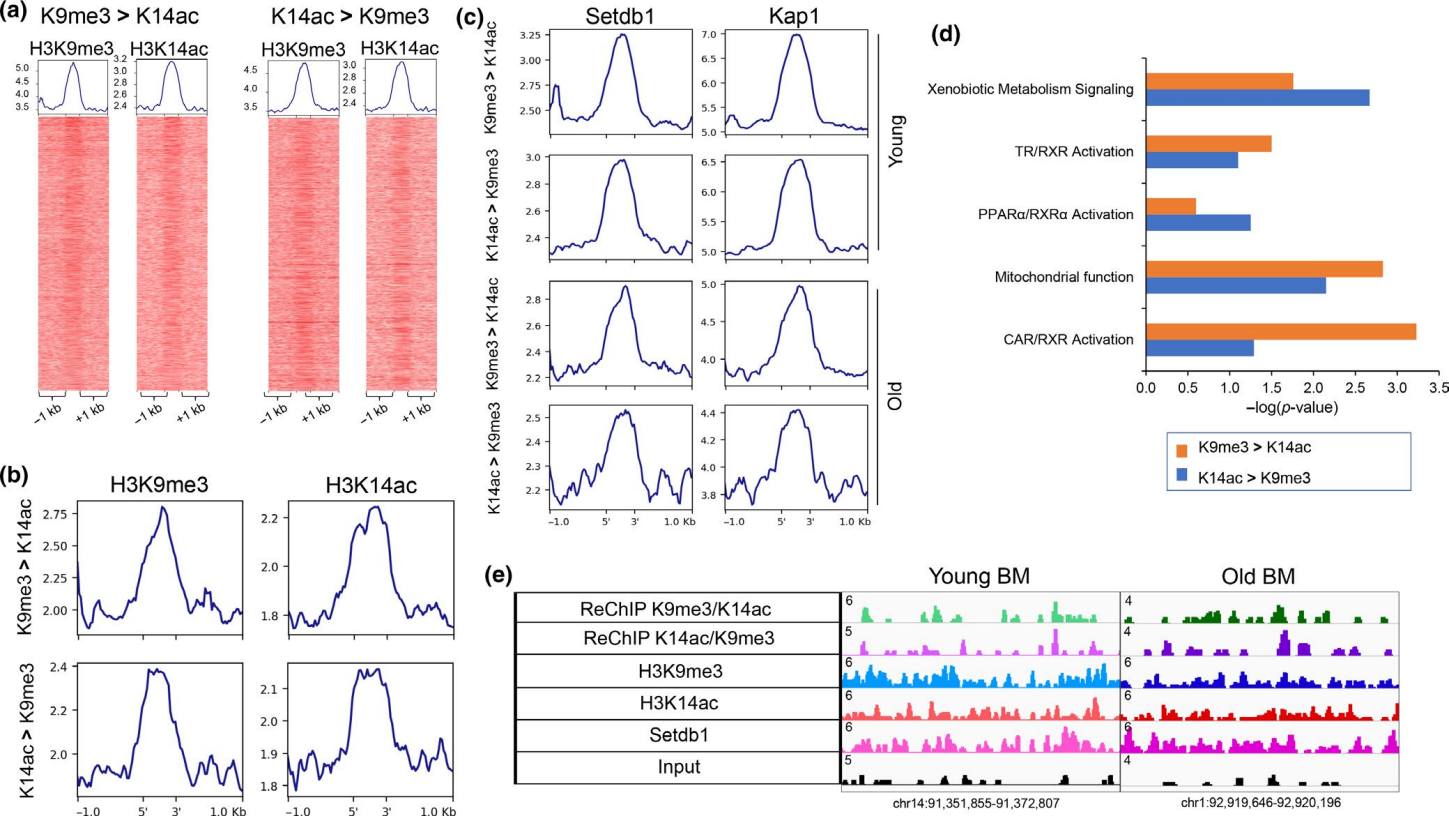
K9me3/K14ac single H3 nucleosomes correlate with H3K9me3/H3K14ac dually marked bivalent genomic regions. (a) Heatmaps showing H3K9me3 (left) and H3K14ac (right) ChIP‐Seq signal at top 5,000 sequential ChIP regions in young livers (H3K9me3 > H3K14ac on the left, H3K14ac > H3K9me3 on the right). (b) Profile plots generates by deeptools showing H3K9me3 (left panel) and H3K14ac (right panel) signal at top 2,000 sequential ChIP regions in old livers (H3K9me3 > H3K14ac, top panel, H3K14ac > H3K9me3, bottom panel). Average number of reads per bin (25 bp) is shown on y‐axis. Reads from one biological replicate in each condition. (c) Profile plots generates by deeptools showing Setdb1 (left panel) and Kap1 (right panel) signal at top 5,000 sequential ChIP regions in young livers (H3K9me3 > H3K14ac, H3K14ac > H3K9me3, top two panels) and at top 2,000 sequential ChIP regions in old livers (H3K9me3 > H3K14ac, H3K14ac > H3K9me3, bottom two panels). Average number of reads per bin (25 bp) is shown on y‐axis. Reads from one biological replicate in each condition. (d) Ingenuity Pathway Analysis of genes that mapped to sequential ChIP regions in young livers (K9me3 > K14ac in orange, K13ac > K9me3 in blue) identified pathways, including activation of nuclear receptors CAR, TR, and PPAR‐dependent gene expression, similar to gene expression changes in old lives. (e) Examples of sequential ChIP regions with bulk H3K9me3 and H3K14ac signal and Setdb1 binding (chr14:91,351,855–91,372,807, left panel and chr1:92,919,646–92,920,196, right panel)

*K9me3/K14ac single nucleosomes correlate with H3K9me3/H3K14ac dually marked bivalent genomic regions.* Our mass spectrometry data showed a quantitative decrease of K9me3/K14ac single H3 histone tails in old livers, while intersection of genomic regions bound by H3K9me3 and H3K14ac identified a comparable number of dually marked genomic sites (1,615 in young and 1,692 in old livers). In order to resolve this issue, we performed sequential ChIP (both H3K9me3 > H3K14ac and H3K14ac > H3K9me3) followed by next‐generation sequencing in young and old livers to identify co‐localization of the two marks at the same genomic locus. We detected both H3K9me3 and H3K14ac bulk signal in reChIP signal, corresponding to dually marked single nucleosomes, in young and old livers that is absent in random genomic regions (Figure 3a,b, Figure S1a,b). The magnitude of reChIP signal is decreased in aged hepatocytes (Figure 3b). In addition, we observed Setdb1 and Kap1 binding in bivalently marked single nucleosome regions, which was absent in random genomic regions (Figure 3c, Figure S1c). The resolution of sequential ChIP can identify bivalent nucleosomes but is not sufficient to pinpoint dually marked single histone tails. However, since we identified Setdb1 ChIP‐Seq signal in sequential ChIP‐Seq regions, a subset of reChIP sites corresponds to dually modified single histone tails because Setdb1 has been shown to recognize and be recruited to dually modified H3K9me3/H3K14ac peptides (Jurkowska et al., 2017). Hence, our results show a correlation between H3K9me3/H3K14ac bulk bivalent regions, bivalent nucleosomes, and dually marked single histone tails. However, pinpointing the exact correspondence between these sites will require further study. Ingenuity Pathway Analysis of genes that mapped to sequential ChIP regions in young livers identified pathways, including activation of nuclear receptors CAR, TR, and PPAR‐dependent gene expression (Figure 3d). similar to gene expression changes in old lives (Figure 2d, (Bochkis et al., 2014). Examples of sequential ChIP regions with bulk H3K9me3 and H3K14ac signal and Setdb1 binding are shown in Figure 3e.

**Figure 4:**
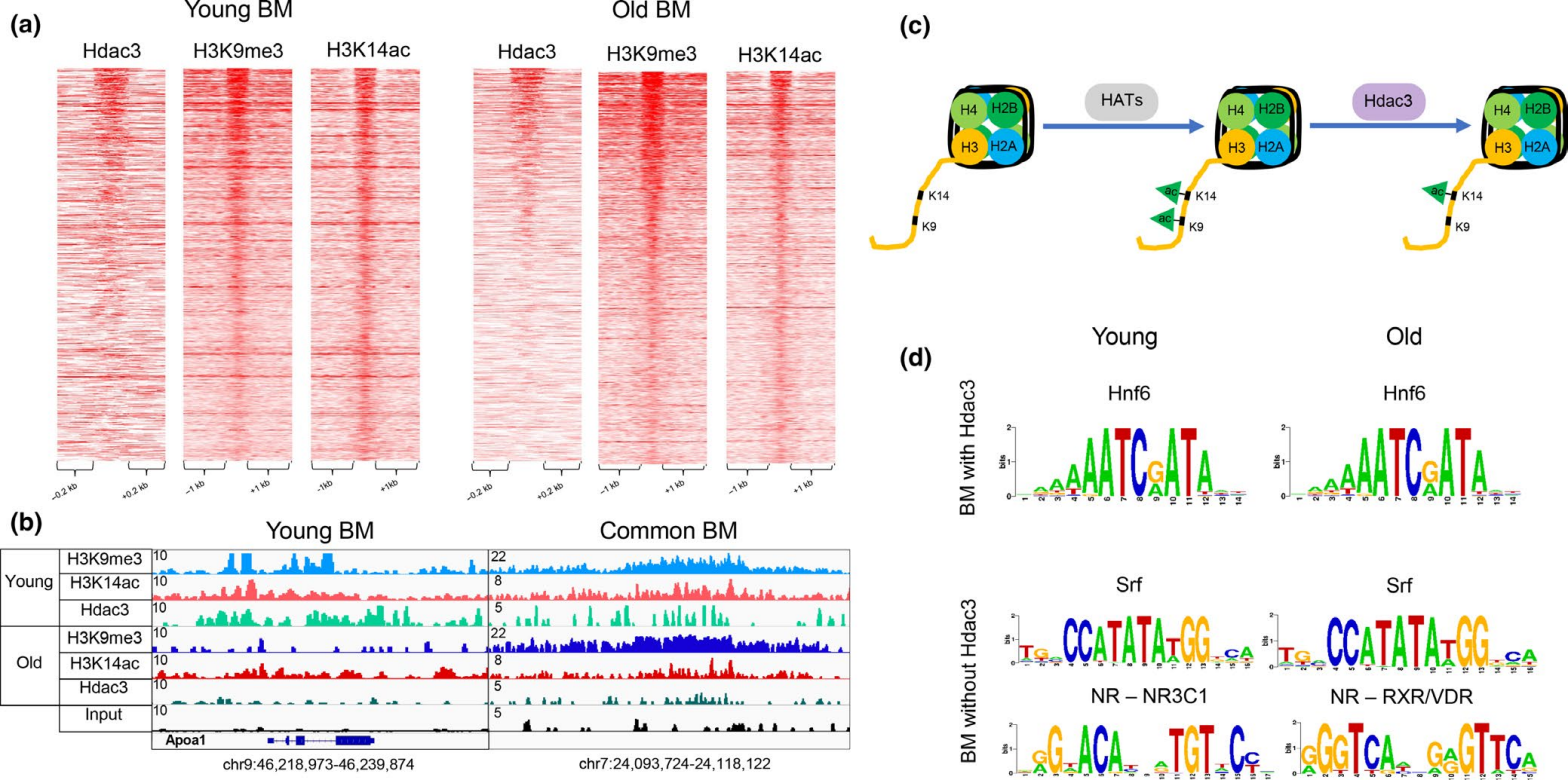
Hdac3 occupies H3K9me3/H3K14ac bivalent regions. (a) Heatmaps showing Hdac3, H3K9me3, and H3K14ac ChIP‐Seq signal at bivalent regions of young (left panel) and old (right panel) livers. A subset of bivalent regions is bound by Hdac3 in both young and old livers. (b) Examples of genomic regions showing the bivalent region bound by Hdac3 in young livers (chr9:46,218,973–46,239,874) and common to young and old livers (chr7:24,093,724–24,118,122). Magnitude of the ChIP‐Seq signal is shown on y‐axis. (c) Model implicating histone deacetylase, Hdac3, in establishment of bivalent mark. First, a histone acetyltransferase acetylates both H3K9 and H3K14, known to be acetylated together in many regions, followed by deacetylation of H3K9 residue by Hdac3, allowing for its subsequent tri‐methylation. (d) Overrepresented motifs in the bivalent regions with and without Hdac3 binding in young and old livers, identified by PscanChIP. Onecut/Hnf6 motifs were enriched in both young and old bivalent regions bound with Hdac3 livers (*p*‐values < 4.3E‐38 and 6.8E‐7, respectively). In contrast, motifs in dually marked regions without Hdac3 occupancy included Srf in both young and old livers (*p*‐values < 0.001 and 6.1E‐6) and several nuclear receptors (young NR3C1, *p*‐value < 7.4E‐7; old RXRA: VDR, *p*‐value < 0.0148). ChIP‐Seq data for Hdac3 (Bochkis et al., 2014) and H3K9me3 (Whitton et al., 2018) are from our previous studies

*Binding of Hdac3, Setdb1, and Kap1 associated with genomic bivalent regions.* We have previously implicated Hdac3, a H3K9 deacetylase, with a role in age‐associated hepatic steatosis (Bochkis et al., 2014). We hypothesized that Hdac3 activity could be important in establishing the dually marked sites. Strikingly, Hdac3 binding sites (ChIP‐Seq, http://www.ncbi.nlm.nih.gov/geo/query/acc.cgi?acc=GSE60393 from (Bochkis et al., 2014)) correlated with presence of H3K9me3 signal in about third of bivalent regions in both young and old livers (Figure 4a). We find that Hdac3 binds bivalent regions that are specific to young livers and common to young and old livers but is not occupying old‐specific bivalent regions. Examples of Hdac3‐bound bivalent regions include apolipoprotein genes in young and inflammatory genes in old livers (Figure 4b, Figure S2a). In addition, we detect Hdac3 binding in dual H3K9me3/H3K14ac reChIP regions young and old livers. The signal is weaker in old livers and is absent in random genomic regions (Figure S2b,c). We propose a model where first a histone acetyltransferase acetylates both H3K9 and H3K14, known to be acetylated together in many regions (Karmodiya et al., 2012), followed by deacetylation of H3K9 residue by Hdac3 allowing for its subsequent tri‐methylation (Figure 4c). Our model is consistent with two previous reports showing that Hdac3 is essential to H3K9ac/H3K9me3 transition (Bhaskara et al., 2010; Ji et al., 2019). Next, in order to differentiate between bivalent regions bound versus not occupied by Hdac3, we performed motif scanning analysis using PscanChIP (Zambelli, Pesole, & Pavesi, 2013). Interestingly, Onecut/HNF6 motif was highly enriched in Hdac3‐bound regions in both young and old livers (*p*‐values < 4.3E‐38 and 6.8E‐7, respectively, Figure 4d). Our analysis is consistent with a previous report implicating HNF6/Hdac3 complex in regulation of hepatic lipid metabolism (Zhang et al., 2016). In contrast, motifs in dually marked regions without Hdac3 occupancy included Srf in both young and old livers (*p*‐values < 0.001 and 6.1E‐6) and several nuclear receptors (young NR3C1, *p*‐value < 7.4E‐7; old RXRA: VDR, *p*‐value < .0148). We have previously identified Srf as a potential regulator of hepatic gene expression responsible for age‐dependent metabolic dysfunction (Bochkis et al., 2014).

**Figure 5:**
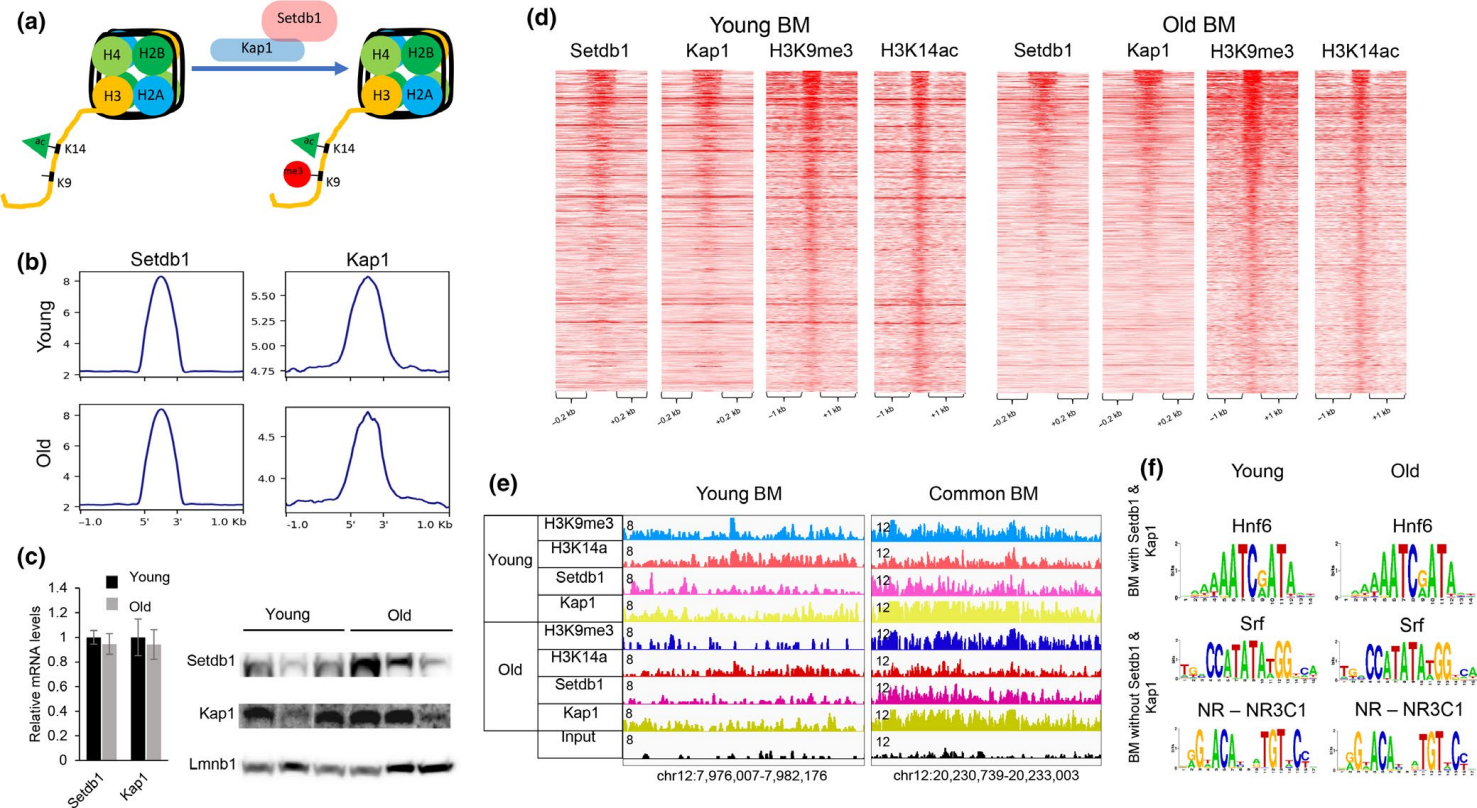
Setdb1/Kap1 complex associates with bivalent regions. (a) Model implicating histone methyltransferase, Setdb1, in establishing regions with bivalent mark. Setdb1, in complex with Kap1, methylates H3K9 by recognizing H3K14 acetylation, creating a new dually marked region. (b) Profile plot generated by deeptools showing Setdb1 (left panel) and Kap1 (right panel) signal at Setdb1‐bound regions in young (top panel) and old (bottom panel) livers. Average number of reads per bin (25 bp) is shown on y‐axis. Reads were merged from two biological replicates in each condition. (c) Relative mRNA levels (*n* = 4, left panel) of Setdb1 and Kap1 by quantitative RT‐PCR. Western blot analysis (*n* = 3, right panel) of protein nuclear extracts with antibodies to Setdb1, Kap1, and Lmnb1 (loading control) in young and old livers. mRNA and protein expression levels of Setdb1 and Kap1 did not change significantly. Gapdh was used as normalizing control in quantitative RT‐PCR. (d) Heatmaps showing Setdb1, Kap1, H3K9me3, and H3K14ac ChIP‐Seq signal at bivalent regions of young (left panel) and old (right panel) livers. A subset of regions with bivalent mark is bound by Setdb1 and Kap1 in both young and old livers. (e) Examples of genomic regions showing bivalent regions bound by Setdb1/Kap1 complex in young (chr12:7,976,007–7,982,176) and common to young and old livers (chr12:20,230,739–20,233,003) livers. Magnitude of the ChIP‐Seq signal is shown on y‐axis. (f) Overrepresented motifs in the bivalent regions with and without Setdb1/Kap1 binding in young and old livers, identified by PscanChIP. Hnf6 motifs were enriched in bivalent regions occupied by Setdb1/Kap1 in both young and old livers (*p*‐values < 1.4E‐34 and < 3.8E‐28, respectively). In contrast, motifs in dually marked regions without Setdb1/Kap1 occupancy included Srf in both young and old livers (*p*‐values < 5.5E‐6 and 0.0088, respectively) and several nuclear receptors (NR3C1, young NR3C1, *p*‐values«.0002 and .0006, respectively). ChIP‐Seq data for H3K9me3 are from our previous study (Whitton et al., 2018)

To complete the assembly of bivalent sites, we propose that deacetylation of H3K9 by Hdac3 is followed by writing of H3K9 triple methylation by a histone methyltransferase Setdb1 (Figure 5a). We identified the overlap of dually marked regions with Kap1 binding sites to be highly significant in both young and old livers (*p*‐value < 7.55E‐10, Figure 2e). Kap1 is often found in complex with Setdb1 (Schultz et al., 2002), a H3K9 methyltransferase associated with H3K14ac/H3K9me3 dual sites in mESCs (Jurkowska et al., 2017). To test the hypothesis, we performed genome‐wide location analysis (ChIP‐Seq) of Setdb1 and Kap1 binding in young and old livers. Setdb1 and Kap1 bind a similar number of sites (50,583, FDR 5%, *q*‐value = 0.04 and 48,915, FDR 5%, *q*‐value = 0.05, respectively) in young livers. Occupancy of both factors is significantly reduced in old livers (13,877 and 22,141, respectively). We identified Kap1 signal at Setdb1 binding sites in both young and old livers (Figure 5b) that is absent in random genomic regions (Figure S3), suggesting that Kap1 occupies Setdb1 genomic regions. Expression of both factors does not change either on mRNA or protein level (Figure 5c). Hence, observed redistribution of binding of these regulators in old livers is independent of expression. Since Setdb1 is associated with H3K9me3/H3K14ac dually marked regions in mESCs (Jurkowska et al., 2017), we looked for presence of Setdb1/Kap1 complex in bivalent regions in young and old livers. Heatmaps of ChIP‐Seq signal show 32% overlap in young (514/1615) and 24% in old (405/1692 hepatocytes (Figure 5d). We find that Setdb1/Kap1 complex binds bivalent regions that are specific to young livers and common to young and old livers but is not occupying old‐specific bivalent regions (Figure 5e). Similar to bivalent regions bound by Hdac3, PscanChIP analysis identified Onecut/HNF6 motif to be significantly enriched in Setdb1/Kap1‐bound bivalent sites in young and old livers (*p*‐values < 1.4E‐34 and < 3.8E‐28, respectively, Figure 5f). In contrast, motifs in dually marked regions without Setdb1//Kap1 occupancy included Srf in both young and old livers (*p*‐values < 5.5E‐6 and 0.0088, respectively) and several nuclear receptors (NR3C1, *p*‐values«.0002 and .0006, respectively).

**Figure 6:**
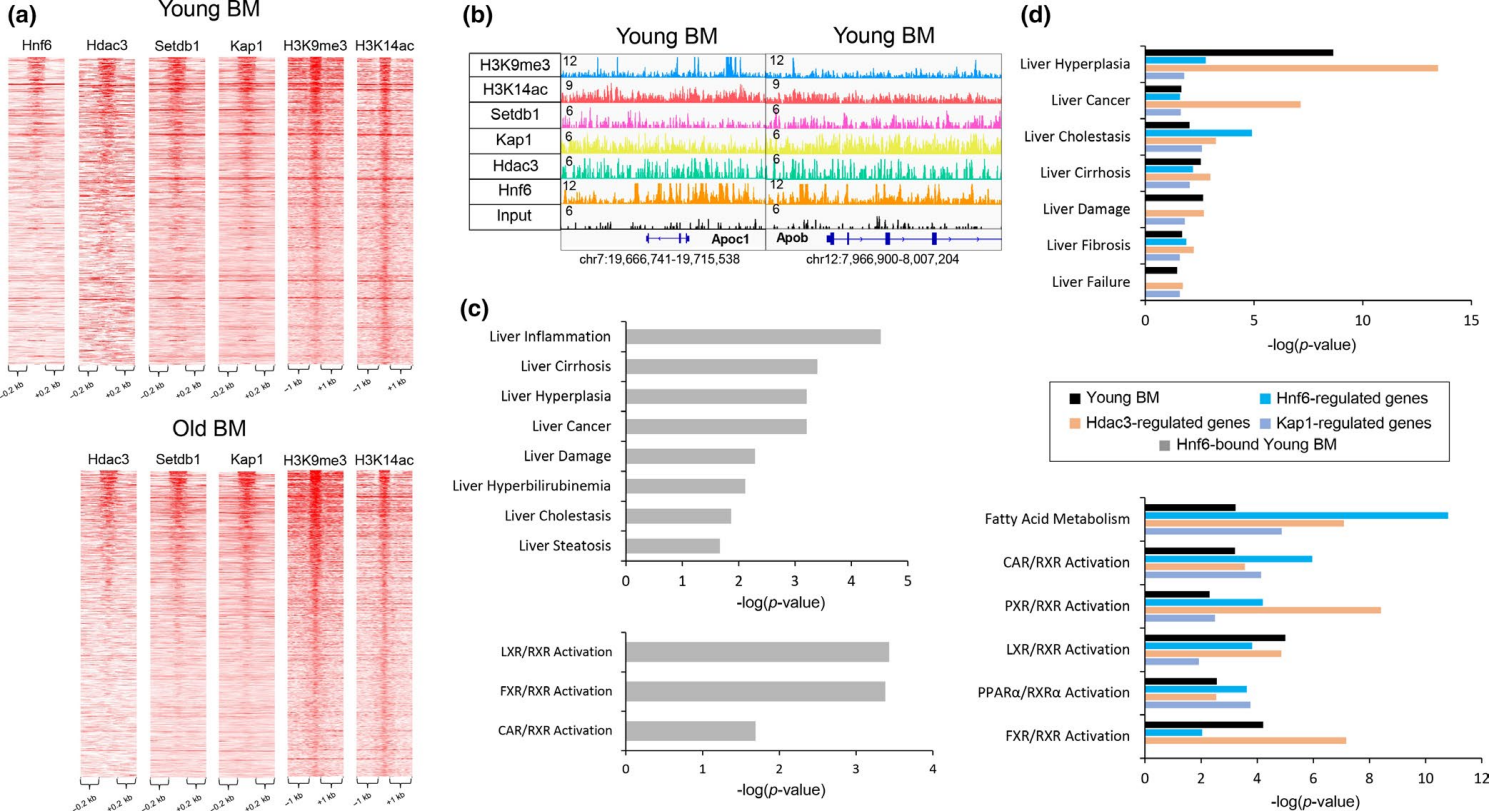
Hdac3, Setdb1, and Kap1 co‐localize to same bivalent regions. (a) Heatmaps showing Hnf6, Hdac3, Setdb1, Kap1, H3K9me3, and H3K14ac ChIP‐Seq signal at regions with bivalent mark in young (left panel) and old livers (right panel). A subset of bivalent regions occupied by both Hdac3 and Setdb1/Kap1 complex is also bound by Hnf6 in young livers. (b) Examples of genomic regions showing the bivalent region bound by Hnf6, Hdac3, and Setdb1/Kap1 complex in young livers include regions near two apolipoprotein genes, Apoc1 (chr7:19,666,741–19,715,538), and Apob (chr12:7,976,007–7,982,176). Magnitude of the ChIP‐Seq signal is shown on y‐axis. (c) Ingenuity Pathway Analysis (IPA) of genes associated with Hnf6‐bound bivalent regions in young livers (Hnf6‐bound Young BM, gray bar) showing significantly overrepresented disease functions (top panel) and pathways (bottom panel). Inflammation, hyperplasia, and steatosis are among most significantly overrepresented hepatotoxicity functions (*p*‐values < 3.92E‐5, 6.24E‐4, 2.16E‐2, and LXR, FXR, and CAR activation pathways (‐log(*p*‐value) 3.34, 3.18) are most significantly overrepresented. (d) Comparison of overrepresented disease functions (top panel) and pathways (bottom panel) identified by Ingenuity Pathway Analysis (IPA) between young liver bivalent mark‐associated genes (Young BM, black bar) and genes regulated by Hnf6 (blue bar), Hdac3 (orange bar), and Kap1 (purple bar). Genes associated with bivalent regions are mapped by Genomic Regions Enrichment of Annotations Tool (GREAT). Expression data for Hnf6 (Zhang et al., 2016), Hdac3 (Sun et al., 2013), and Kap1‐regulated (Bojkowska et al., 2012) gene sets are published microarray studies. ChIP‐Seq data for Hnf6 are from a published study (Wang et al., 2014) and for H3K9me3 are from our previous study (Bochkis et al., 2014)

*Hdac3, Setdb1, and Kap1 co‐localize to same bivalent regions.* Considering that both Hdac3‐bound and Setdb1/Kap1 bound bivalent regions were enriched for the sample Onecut/HNF6 motif, we next investigated the possibility if Hdac3 and Setdb1/Kap1 complex were occupying the same sites. Remarkably, the three factors were bound in same regions in both young and old livers (Figure 6a). Our observations are consistent with a previous report showing that N‐Cor complex containing Hdac3 interacts with Kap1 (Underhill, Qutob, Yee, & Torchia, 2000). Next, we wanted to ascertain whether HNF6 was indeed bound in bivalent regions as motif analysis suggested. We compared HNF6 binding in young liver from a previous study (ArrayExpress E‐MTAB‐2060)(Wang et al., 2014) finding that occupies a subset of Hdac3/Setdb1/Kap1 bound bivalent sites (19% overlap, 308/1615 of all bivalent sites in young liver, Figure 6a). Examples of HNF6 bound elements at apolipoprotein genes Apob and Apoc1 are shown in Figure 6b. Subsequent comparison of genes in HNF6‐bound bivalent regions in young livers and differentially expressed genes in old livers showed an overlap of pathways, including activation of CAR, FXR, and LXR‐dependent gene expression (‐log(*p*‐value) 1.69, 3.18, 3,34, IPA, Figure 6c). Further, we compared genes differentially expressed in HNF6, Hdac3, and Kap1 knockout livers with genes whose expression changed in old hepatocytes. Remarkably, the overlap included similar pathways enriched in all three knockout models. These include activation of LXR and PPAR‐dependent gene expression, consistent with development of steatosis in aged liver (Figure 6d).

**Figure 7:**
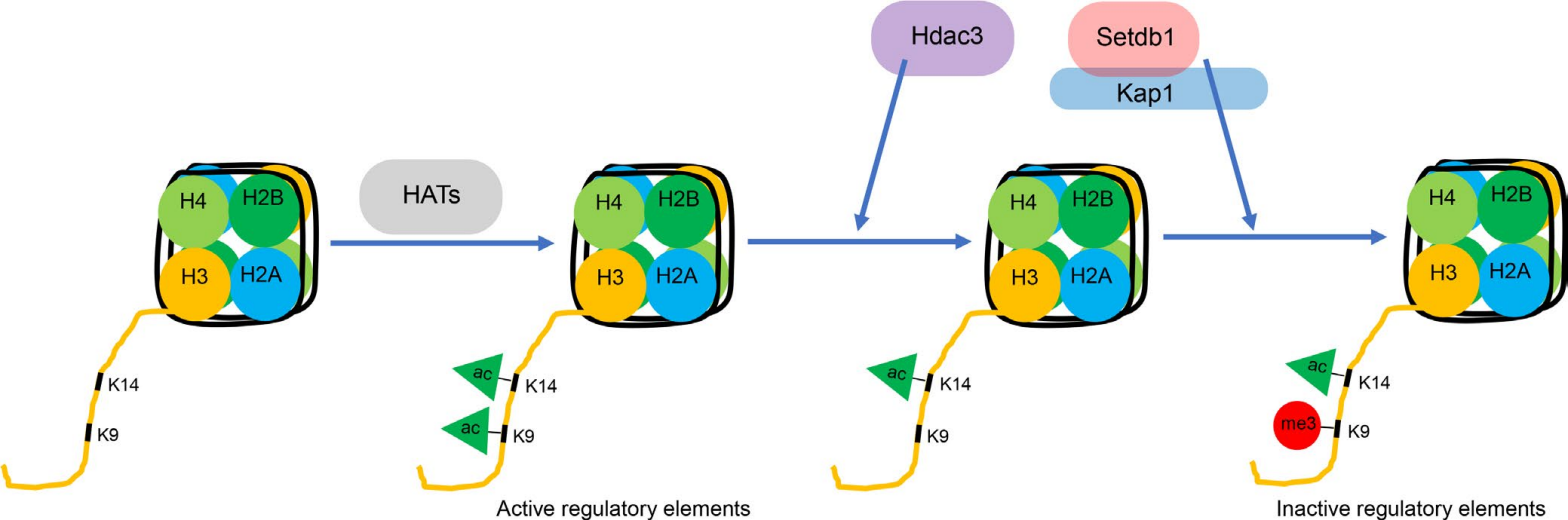
Model describing establishment of H3K9me3/H3K14ac bivalent mark. We show that Hdac3, Setdb1, and Kap1 co‐localize to both single histone tail and bulk dually marked H3K9me3/H3K14ac genomic regions. This bivalent mark is established in two steps. First, close residues H3K9 and H3K14 are acetylated by the same histone acetyltransferase (HAT). And second, H3K9 is deacetylated by Hdac3, allowing for subsequent triple methylation of H3K9 by Setdb1, which is found in complex with Kap1, and establishment of the bivalent region. There is a possibility that Hdac3 and Setb1 could be in the same complex. Hdac3 is a part of Ncor1 complex that has been shown to interact with Kap1 (Underhill et al., 2000), which in turn interacts with Setdb1 (Schultz et al., 2002)

In summary, we show a correlation between H3K9me3/H3K14ac dually marked single histone tails and H3K9me3/H3K14ac bulk bivalent genomic regions (Figure 1a, Figure 3a). We propose a model where first close residues H3K9 and H3K14 are acetylated by the same histone acetyltransferase (HAT), then H3K9 is deacetylated by Hdac3, allowing for subsequent triple methylation of H3K9 by Setdb1/Kap1 and establishment of the bivalent region (Figure 7). Our model is consistent with a prior study reporting that, first, established H3K14ac mark is recognized by the Tudor domain of Setdb1, followed by triple methylation of H3K9 by the SET domain of Setdb1 (Jurkowska et al., 2017).

## 3 DISCUSSION

Here, we performed chromatin profiling by quantitative targeted mass spectrometry to assess all possible modifications of the core histones in young and old liver. We discovered that a hepatic bivalent combination, dually marked H3K9me3/H3K14ac modification, is significantly decreased in old hepatocytes. Subsequent sequential ChIP‐Seq identified dually marked single nucleosome regions, with reduced number of sites and decreased signal in old livers, confirming mass spectrometry results. We detected H3K9me3 and H3K14ac bulk ChIP‐Seq signal in reChIP single nucleosome regions, suggesting a correlation between H3K9me3/H3K14ac bulk bivalent genomic regions and dually marked single nucleosomes. Presence of this bivalent mark has been reported previously in mESCs where the authors linked H3K14ac, recognized by the Tudor domain of Setdb1, to H3K9me3, methylated by SET domain of Setdb1 (Jurkowska et al., 2017). The authors reported a larger H3K14ac/H3K9me3 overlap in ES cells (12,400 bivalent regions, 2,231 bound by Setdb1). We observe a smaller number of bivalent genomic regions in adult differentiated tissue.

In addition to Setdb1, we also localized Kap1, an interaction partner of Setdb1, and Hdac3, an enzyme that deacetylates H3K9, to bivalent regions. While binding of Hdac3 is typically associated with absence of H3K9ac mark (Feng et al., 2011), we find that Hdac3 correlates with presence of H3K9me3 modification. We propose that Hdac3/Setdb1/Kap1 act in concert, with Hdac3 first deacetylating H3K9, followed by subsequent triple methylation by Setdb1. About half of sites occupied by Hdac3/Setdb1/Kap1 are also bound by HNF6. Hdac3 has been shown to bind DNA in complex with HNF6 (Zhang et al., 2016) at regulatory elements of lipid metabolic genes but a relationship between HNF6 and Setdb1 has not been reported previously. Since Hdac3 binding is circadian (Feng et al., 2011), it is likely that a different subset of HNF6‐bound regions could overlap with Hdac3 at a different time point.

Further, we observe similarities between genes connected to bivalent marks in young liver and those differentially expressed in old liver. Apolipoprotein genes that govern cholesterol export and genes important for triglyceride synthesis are of particular interest. They correspond to gene expression and metabolic changes, such as increased cholesterol secretion (Einarsson, Nilsell, Leijd, & Angelin, 1985) and development of hepatic steatosis (Whitton et al., 2018) in old livers. Interestingly, these genes are connected to regions bound by HNF6, Hdac3, and Setdb1/Kap1 and comparison analysis of genes regulated by these factors identified pathways similar to those upregulated in old livers. Together, these observations suggest that H3K9me3/H3K14ac dually marked regions constitute a poised inactive state in young livers, which is resolved in old livers, leading to changes in gene expression that contribute to metabolic dysfunction.

## 4 EXPERIMENTAL PROCEDURES

### 4.1 Mice

Young (3 months) and old (21 months) male mice (C57BL/6) were purchased from the National Institute of Aging (NIA) aged rodent colony (Charles River Laboratories). Upon arrival, animals were housed for a week to acclimate to the light‐dark cycle at the UVa facility before tissue harvest. Four biological replicates of young and old mice were used for proteomics study. All animal work was approved by Animal Care and Use Committee at UVa (protocol number 4162–03–17).

### 4.2 Analysis of histone modifications by targeted mass spectrometry

Quantitative targeted mass spectrometry‐based profiling of histone modifications was performed at LINCS Proteomic Characterization Center for Signaling and Epigenetics at The Broad Institute of MIT and Harvard University, Cambridge, MA. Detailed experimental procedure for the complete proteomics study is described previously (Creech et al., 2015). Histone modification profiling was performed using the mass spectrometry‐based Global Chromatin Profiling (GCP) assay, as described in detail previously (Creech et al., 2015). Tissue samples were dissected into ~ 1 mm^3^ pieces with razor blades and homogenized in nucleus buffer (as in (Creech et al., 2015)) with an electronic pestle fitted into a microcentrifuge tube. Acid extracted histone preparations were purified by TCA precipitation, mixed 1:1 by mass with 13C6,15N4‐arginine SILAC‐labeled histones derived from an equal parts mixture of HeLa, 239T, and K562 cells, derivatized with NHS‐propionate, digested by trypsinization, rederivatized, and modification profiles were generated by mass spectrometric measurements between the endogenous liver peptides and the SILAC‐labeled standards. All samples were separated on an online Proxeon EASY‐nLC 1,000 UHPLC system (Thermo Scientific) and analyzed on a Q Exactive mass spectrometer (Thermo Scientific). Heatmap of relative abundance of histone modifications was drawn by Morpheus (Morpheus, https://software.broadinstitute.org/morpheus). Analysis of mass spectrometry data was performed as described previously (Creech et al., 2015).

### 4.3 RNA and protein analysis

Analysis of mRNA and protein expression levels were performed as described previously (Whitton et al., 2018). Gapdh was used as normalizing gene for quantitative RT‐PCR analysis. Primer sequences will be provided upon request. Lmnb1 (Abcam, ab16048) was used as loading control. Rabbit polyclonal antibodies specific to KAP1 (Abcam, ab10483) and SETDB1 (Proteintech, 11231–1‐AP) were used in western blotting at 1:1,000 dilution. Student's two sample *t* test was used to analyze the Q‐PCR data.

### 4.4 Chromatin immunoprecipitation, sequential ChIP, and sequencing

Snap‐frozen mouse liver (100 mg) from young and old wild‐type mice was used to prepare chromatin. ChIP and H3K14ac ChIP‐Seq were performed as described previously (Whitton et al., 2018). For Setdb1 and Kap1, a few modifications were incorporated. Particularly, sonication, using Diagenode Bioruptor Pico, was reduced to 11 cycles (30 s pulse on, 30 s pulse off) and libraries were sequenced using Illumina NextSeq 500 following the manufacturer's protocols. Rabbit polyclonal antibodies specific to KAP1 (Abcam, ab10483), SETDB1 (Proteintech, 11231–1‐AP), and anti‐acetyl‐histone‐H3 specific to Lys‐14 (Millipore, 07–353) were used for immunoprecipitation.

For sequential ChIP, the first immunoprecipitation was performed as described for ChIP (Whitton et al., 2018) with some modifications. After the washes, samples were eluted in 100 µl 1% SDS and 10 mM fresh dithiothreitol (DTT) twice for a total elution volume of 200 µl. After each elution, the samples were rotated at 37ºC for 15 min. After the second elution, samples were diluted in TE to 1.5 ml. Then, the second antibody was added and the second ChIP was performed as described previously (Whitton et al., 2018). Rabbit antihistone H3 (trimethyl K9) antibody (ab8898) and anti‐acetyl‐histone‐H3 specific to Lys‐14 (Millipore, 07–353) were used for immunoprecipitation.

### 4.5 ChIP‐Seq and sequential ChIP‐Seq analysis

Detailed description of in silico analysis of sequenced reads is previously described (Whitton et al., 2018). Briefly, reads were aligned to the mouse genome (mm10; NCBI Build 38) using BWA v0.7.12(Li & Durbin, 2009). Duplicate reads were removed using Picard v 1.134 (http://picard.sourceforge.net). Reads (phred score ≥30) that aligned uniquely were used for subsequent analysis. Data from two biological replicates were merged for Kap1 ChIP‐Seq comparison (2 Young and 2 Old). Data from three biological replicates were merged for H3K14ac and Setdb1 ChIP‐Seq comparison (3 Young and 3 Old). PeakSeq (Rozowsky et al., 2009) was used to identify bound peaks against input controls (H3K14ac: FDR 5%, *q*‐value = 0.02; Setdb1: FDR 5%, *q*‐value = 0.04, Kap1: FDR 5%, *q*‐value = 0.05). We also used PeakSeq to call peaks in previously published H3K9me3 data (http://www.ncbi.nlm.nih.gov/geo/query/acc.cgi?acc=GSE78177, FDR 5%, *q*‐value = 0.01). We used published results for Hdac3 data (Bochkis et al., 2014) ((http://www.ncbi.nlm.nih.gov/geo/query/acc.cgi?acc=GSE60393, FDR 5%, *q*‐value = 0.2).

PeakSeq was also used to identify peaks in sequential ChIP‐Seq data. Top 5,000 regions in young livers and top 2,000 regions in old livers (both H3K9me3 > H3K14ac reChIP and H3K14ac > H3K9me3 reChIP) were used for subsequent analysis.

### 4.6 Functional analysis

Sequencing reads were visualized with the Integrative Genome Viewer (IGV)(Robinson et al., 2011). Ingenuity Pathway Analysis (IPA) and PWM scan analysis using PscanCHIP (assembly: mm10, background: mixed) were performed as reported previously (Bochkis et al., 2014). Reads were extended to the size of the library insert (150 bp) to obtain sequences for motif analysis. Functional analysis of ChIP‐Seq peaks was performed as described previously (Whitton et al., 2018) with the following modifications. ChIP‐Seq peaks were associated with closest genes by Genomic Regions Enrichment of Annotations Tool (GREAT) with basal plus extension method and distal regions extended up to 100,000 kb (McLean et al., 2010). Overlap of ChIP‐Seq peak regions was analyzed by bedtools (intersect intervals tool using default parameters) in Galaxy (Hillman‐Jackson et al., 2012). Heatmaps of ChIP‐Seq coverage were generated by deeptools (Ramirez, Dundar, Diehl, Gruning, & Manke, 2014). Expression data from published studies for Hdac3 (http://www.ncbi.nlm.nih.gov/geo/query/acc.cgi?acc=GSE49386), Hnf6 (http://www.ncbi.nlm.nih.gov/geo/query/acc.cgi?acc=GSE83789), and Kap1‐regulated genes (Bojkowska et al., 2012) were accessed from GEO. Differentially expressed genes from published microarray studies were identified by GEO2R tool(Barrett et al., 2013). Chromatin‐x enrichment analysis and analyses for overrepresented pathways and disease conditions were performed by Enrichr (Kuleshov et al., 2016).

### 4.7 Accession numbers

ChIP‐Seq data from this study can be accessed at GEO under accession numbers http://www.ncbi.nlm.nih.gov/geo/query/acc.cgi?acc=GSE130712 for H3K14ac, Setdb1, and Kap1, http://www.ncbi.nlm.nih.gov/geo/query/acc.cgi?acc=GSE60393 for Hdac3, http://www.ncbi.nlm.nih.gov/geo/query/acc.cgi?acc=GSE78177 for H3K9me3, respectively, and ArrayExpress experiment E‐MTAB2060 for Hnf6.

## CONFLICT OF INTEREST

Authors have nothing to report.

## AUTHOR CONTRIBUTIONS

A.J.P. analyzed data and developed a new direction for the project, M.C.M. performed experiments and data analysis, J.K. and S.A. analyzed the data, and I.M.B. developed the project, performed experiments and data analysis, and wrote a draft of the manuscript.

## Data Availability

ChIP‐Seq data from this study can be accessed at GEO under accession numbers http://www.ncbi.nlm.nih.gov/geo/query/acc.cgi?acc=GSE130712 for H3K14ac, Setdb1, and Kap1, http://www.ncbi.nlm.nih.gov/geo/query/acc.cgi?acc=GSE60393 for Hdac3, http://www.ncbi.nlm.nih.gov/geo/query/acc.cgi?acc=GSE78177 for H3K9me3, respectively, and ArrayExpress experiment E‐MTAB2060 for Hnf6.
