# Supplementary material for: Hdac3, Setdb1, and Kap1 mark H3K9me3/H3K14ac bivalent regions in young and aged liver
Source: Aging Cell. 2019 Dec 19;19(2):e13092. doi: 10.1111/acel.13092 (PMC6996956; doi:10.1111/acel.13092)

Supplementary Information for

Hdac3, Setdb1, and Kap1 mark H3K9me3/H3K14ac bivalent regions in young and aged liver.

Andrew J. Price, Mohan C. Manjegowda, Jessica Kain, Swetha Anandh, Irina M. Bochkis

Supplementary Figure Legends

Supplementary Table 1 Quantitative targeted Mass-Spectrometry-based profiling of histone modifications Table contains both raw, processed, and final data values for the mass spectrometry experiment.

Supplementary Figure 1 (**a**) Heatmaps showing H3K9me3 (left) and H3K14ac (right) ChIP-Seq signal at random 5,000 regions in young livers (H3K9me3>H3K14ac on the left, H3K14ac>H3K9me3 on the right), corresponding to regions in **Figure 3a**. (**b**) Profile plots generated by deeptools showing H3K9me3 (left panel) and H3K14ac (right panel) signal at random 2,000 sequential ChIP regions in old livers (H3K9me3>H3K14ac, top panel, H3K14ac>H3K9me3, bottom panel), corresponding to regions in **Figure 3b**. Average number of reads per bin (25 bp) is shown on y-axis. Reads from one biological replicate in each condition. (**c**) Profile plots generated by deeptools showing Setdb1 (left panel) and Kap1 (right panel) signal at random 5,000 sequential ChIP regions in young livers (H3K9me3>H3K14ac, H3K14ac>H3K9me3, top two panels) and at random 2,000 sequential ChIP regions in old livers (H3K9me3>H3K14ac, H3K14ac>H3K9me3, bottom two panels), corresponding to regions in **Figure 3c**. Average number of reads per bin (25 bp) is shown on y-axis Reads from one biological replicate in each condition.

Supplementary Figure 2 (**a**) IGV plot of genomic regions where Hdac3 localizes to H3K9me3/H3K14ac bivalent genomic regions in young livers (top left: chr19:44,406,499-44,407,786, top right: chr5:90,460,368-90,461,958, bottom left: chr9:46,228,244-46,230,855, bottom right: chr12:7,977,226-7,978,484). (**b**) Profile plots generated by deeptools showing Hdac3 signal at top 5,000 sequential ChIP regions in young livers (left, top panel: H3K9me3>H3K14ac, bottom panel: H3K14ac>H3K9me3) and at top 2,000 sequential ChIP regions in old livers (right,

top panel: H3K9me3>H3K14ac, bottom panel: H3K14ac>H3K9me3). Average number of reads per bin (25 bp) is shown on y-axis Reads from one biological replicate in each condition. (**c**) Profile plots generated by deeptools showing Hdac3 signal at random 5,000 regions in young livers (left, top panel: H3K9me3>H3K14ac, bottom panel: H3K14ac>H3K9me3) and random 2,000 regions in old livers (right, top panel: H3K9me3>H3K14ac, bottom panel: H3K14ac>H3K9me3), corresponding to sequential ChIP regions in (**b)**. Average number of reads per bin (25 bp) is shown on y-axis Reads from one biological replicate in each condition.

Supplementary Figure 3 Profile plot generated by deeptools showing Setdb1 (left panel) and Kap1 (right panel) signal at random regions in young (top panel) and old (bottom panel) livers, corresponding to Setdb1-bound genomic regions in **Figure 5b**. Average number of reads per bin (25 bp) is shown on y-axis Reads were merged from two biological replicates in each condition.

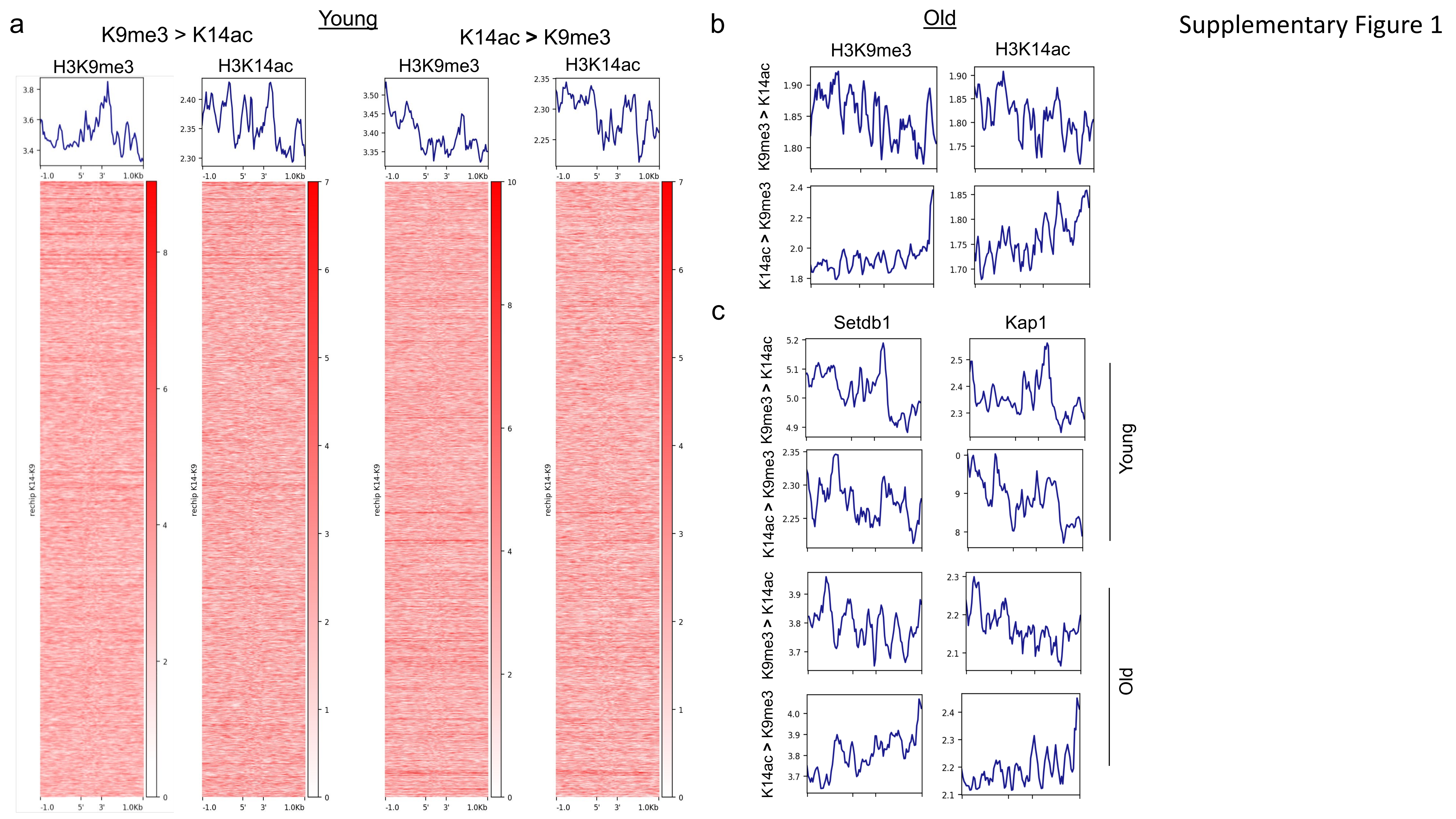

Supplementary Figure 1
a
Young
K9me3 > K14ac
K14ac > K9me3
H3K9me3
H3K14ac
H3K9me3
H3K14ac
rechip K14-K9
-1.0
5'
3'
1.0Kb
b
Old
H3K9me3
H3K14ac
K9me3 > K14ac
K14ac > K9me3
c
Setdb1
Kap1
K9me3 > K14ac
K14ac > K9me3
K9me3 > K14ac
K14ac > K9me3
Young
Old

Supplementary Figure 2

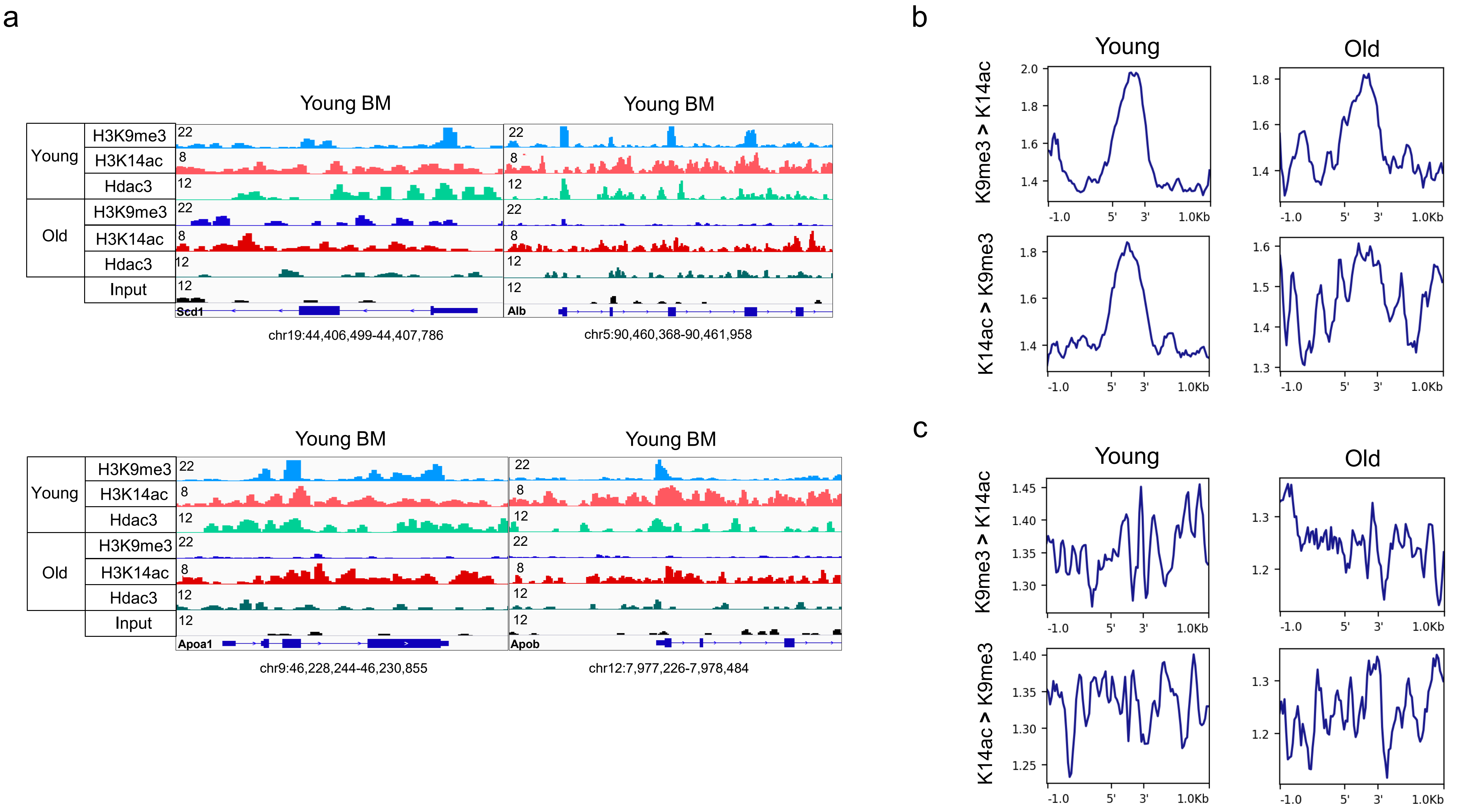

Supplementary Figure 3

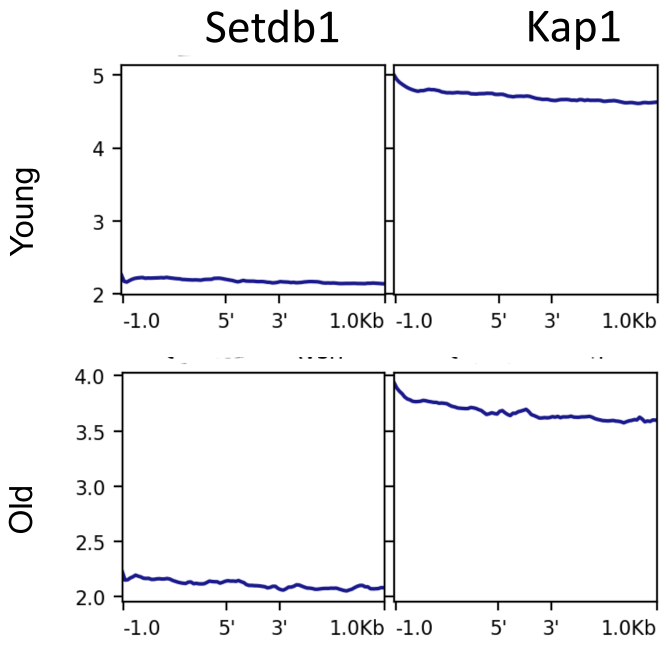

Supplement: Supplementary file 1 [file ACEL-19-e13092-s001.pdf]
